# Drug utilization and cost associated with inflammatory bowel disease management in Saudi Arabia

**DOI:** 10.1186/s12962-019-0194-3

**Published:** 2019-12-04

**Authors:** Yazed AlRuthia, Othman Alharbi, Abdulrahman M. Aljebreen, Nahla A. Azzam, Majid A. Almadi, Ohud H. Bahari, Khalid A. Almalki, Abdulaziz T. Atham, Ahmed S. Alanazi, Maria Saeed, Baraa HajkhderMullaissa, Mohammad Alsenaidy, Bander Balkhi

**Affiliations:** 1 Department of Clinical Pharmacy, College of Pharmacy, King Saud University, P.O. Box 2454, Riyadh, 11451 Saudi Arabia; 2 Pharmacoeconomics Research Unit, College of Pharmacy, King Saud University, Riyadh, Saudi Arabia; 3 Gastroenterology Division, Department of Medicine, King Khalid University Hospital, King Saud University, Riyadh, Saudi Arabia; 4 Division of Gastroenterology, The McGill University Health Center, Montreal General Hospital, McGill University, Montreal, Canada; 5 Department of Pharmaceutics, College of Pharmacy, King Saud University, Riyadh, Saudi Arabia

**Keywords:** Inflammatory bowel diseases, Crohn disease, Colitis, ulcerative, Drug utilization

## Abstract

**Background:**

There has been an increase in incidence and prevalence of inflammatory bowel disease (IBD) outside the western countries. Treatment costs are an essential component for healthcare planning and priority setting. The utilization patterns and annual administration and cost of IBD medications are largely unknown in countries with an increasing incidence of disease, Saudi Arabia being an example.

**Aim:**

To evaluate the use of non-biologic and biologic agents and their associated annual administration costs in a sample of patients with Crohn’s disease (CD) and ulcerative colitis (UC) in Saudi Arabia.

**Methods:**

Single-center retrospective chart review was performed to determine the use of biologic and non-biologic medications among IBD patients in a tertiary care hospital in Riyadh, Saudi Arabia. Daily and the annual acquisition cost of different IBD therapeutic agents was calculated. The utilization rates and cost of each type of medication by CD and UC patients were compared.

**Results:**

Data of 258 CD patients and 249 UC patients were analyzed. Infliximab and adalimumab were the most commonly prescribed biologics among the study sample, however, their utilization rates were significantly higher among CD than UC patients (36.82% vs. 11.24%, and 20.54% vs. 9.64%, respectively, *P *< 0. 01). Azathioprine utilization rate was also higher among CD patients compared to their UC counterparts (71.71% vs. 40.16%, respectively, *P *< 0.01). However, the utilization rate of mesalazine in the UC patients was significantly higher than their CD counterparts (85.53% vs. 14.34% for CD, *P *< 0.01). The annual cost of biologics (including administration and lab test cost) ranged from 5572 USD for ustekinumab to 18,424 USD for vedolizumab. On the other hand, the annual cost of non-biologics ranged from 16 USD for prednisone to 527 USD for methotrexate.

**Conclusion:**

Biologics are extensively used in the management of IBD, particularly CD, and their utilization costs are significantly higher than non-biologics. Future studies should examine the cost effectiveness of IBD medications especially in countries with increasing incidence such as Saudi Arabia.

## Background

The inflammatory bowel disease (IBD) is an idiopathic autoimmune condition which typically presents in two forms: Crohn’s disease (CD) and ulcerative colitis (UC) [1, 2]. It is commonly diagnosed among patients between 15 and 40 years of age, and associated with immense negative impact on patients’ health-related quality of life (HRQoL) as well as on the rate of health care services utilization [3, 4]. The prevalence of IBD is believed to be the highest in the Western world [5]. Its incidence in the United States ranges from 6 to 12.6 cases per 100,000 person-years [6], and reaches almost 30 cases per 100,000 person-years in Australia [7]. Moreover, it is estimated that more than 1.5 million individuals suffer from IBD in North America, and 2 million individulas in Europe [5], and almost 30,000 new cases are being reported annually in that part of the world [8]. However, the incidence of both UC and CD is increasing as well in other parts of the world particularly in newly industrialized countries [5]. Although the IBD-related hospitalization rate has been stable over the past few years in North America and Europe, it is rapidly increasing in the developing countries [9].

In Saudi Arabia, the prevalence and incidence of IBD are largely unknown. A single published national multicenter study on IBD, carried out from 2003 to 2012, determined that the incidence rate among pediatric patients (0–14 years) was 0.2, 0.27, and 0.47 per 100,000 individuals for UC, CD, and IBD, respectively [10]. Another investigation, based on a retrospective review of medical records from 1983 to 2002 in a single center, established that the annual incidence rate was 0.94 per 100,000 individuals [11]. Additionally, the data on 693 IBD patients who have been seen over a period of 17 years in a single medical center have shown that approximately 34% of them were affected by UC and 66% by CD, and most of them were male [12]. However, a smaller single-center study did not reveal sex differences in IBD prevalence among Saudi patients [13]. In another study that explored the epidemiologic, clinical, and phenotypic charactersitics of UC patients in four tertiary care hospital in three cities in Saudi Arabia, no significant difference in the percentages of male and female patients affected by UC was noted and the majority of UC patients were young (e.g., 17–40 years of age) [14]. Likewise, the majority of CD patients were young (e.g., 17–40 years of age) according to a multi-center study that explored the characteristics of CD patients in Saudi Arabia, however, the majority of patients were male [15].

The pharmacologic management of UC and CD is broadly similar and aims at inducing and then maintaining remission [2, 16–18]. The treatment typically centers on therapeutic agents suppressing the inflammatory process, which can be classified as either biologic or non-biologic agents. The biologic agents are represented by monoclonal antibodies and include inactivating antibodies directed against tumor necrosis factor-α (e.g., infliximab, adalimumab, certolizumab, and golimumab), interleukins 12 and 23 (e.g., ustekinumab, brazikumab, and risankizumab), or α4-integrin (e.g., natalizumab and vedolizumab). The non-biologic agents include glucocorticoids (e.g., prednisone, budesonide), aminosalicylates (e.g., sulfasalazine and 5-aminosalicylic acid), calcineurin inhibitors (e.g., cyclosporine and tacrolimus), antimetabolites (e.g., azathioprine and methotrexate), Janus kinase (JNK) inhibitors (e.g., tofacitinib, filgotinib, and upadacitinib), and sphingosine-1-phosphate receptor modulators (e.g., ozanimod) [18, 19]. Over the last decade, biologics have been proven to effectively induce mucosal healing and improve the health-related quality of life (HRQoL) in both UC and CD patients [20–22]. These encouraging results promoted an increasing trend in the utilization of biologics [23, 24]. From 2007 to 2015, the USA market share of biologics for IBD according to the Truven Marketscan Commercial Claims and Encounters increased from 21.8 to 43.8% for CD patients, and from 5.1 to 16.2% for UC patients. During the same period, the rate of utilization of non-biologic drugs, such as 5-ASA, did not increase [24]. Data obtained in Central and Eastern European countries indicated that while the use of biologics to treat IBD increased, their utilization rate was lower among UC compared to CD patients despite a higher prevalence of UC [23].

The cost burden of IBD is substantial [25, 26]. Although traditionally the IBD has been treated using less expensive non-biologic agents, the advent of biologics has revolutionized the treatment of IBD but also increased the expenses on the health systems [19]. It is estimated that the annual cost of illness for IBD exceeds 7 billion United States Dollars (USD) in North America and 5 billion euros in Europe [25–27]. However, with the emergence of IBD in newly industrialized countries and the use of biologics this cost has become substantially higher and is expected to grow further [24, 26]. In fact, the cost of biologics has surpassed other IBD-related costs such as hospitalization and surgery. A Dutch study documented that the cost of anti-TNFα biologics markedly exceeded the cost of hospitalization, surgery, and loss in productivity in both CD and UC patients [28]. In the USA, the cost of biologics for CD patients comprised nearly 30% of the total health care expenses, exceeding inpatient care costs which accounted for only 23% [29].

The data on the utilization patterns and economic burden of IBD medications in new incidence regions is scarce. In Saudi Arabia, the only published study used the data collected from a sample of 312 patients between 1970 and 2008, i.e., a time when biologics were not available or only began to enter the medical armamentarium for IBD treatment. Only infliximab and adalimumab were reported to be used among the study sample, and their combined utilization rates were 21% for CD and 4% for UC [13]. Given the expected shift in the structure of IBD-related health care expenditures with the higher utilization rate of biologics, this study aimed to explore the current biologic and non-biologic utilization patterns and costs among a sample of IBD patients in Saudi Arabia.

## Methods

The current study was designed as a single-center, retrospective chart review of treatment patterns among IBD patients. Data for the period from March 2016 to October 2018 were retrieved from the electronic medical records of a university-affiliated tertiary care hospital in Riyadh, Saudi Arabia. A list of electronic medical record numbers of all patients with a confirmed diagnosis of IBD, either UC or CD, were provided by the department of medicine. Both inpatient and outpatient electronic health records were reviewed. Patients who were not prescribed and dispensed any medications for IBD were excluded. The prices of IBD medications were retrieved from the online database of drug prices maintained by the Saudi Food and Drug Authority (SFDA). The daily cost of each medication was calculated based on the World Health Organization (WHO) Defined Daily Dose (DDD), which was developed as a universal measure of drug consumption and provides a rough estimate of the utilization of different medications used for different indications including IBD. The annual acquisition costs for the identified medications in the patients’ electronic health records were estimated using the DDD due to the lack of data on the actual consumption rate of different medications for IBD at a national level in Saudi Arabia [30]. The cost of laboratory tests, imaging exams, intravenous infusions, nursing fees, and other relevant items was obtained from the Cost Center in the Department of Resources at the Saudi Ministry of Health. Finally, the annual administration cost of each biologic for the first year of treatment was calculated based on the guidelines and protocols of the British Society of Gastroenterology and National Health Service (England), which are the followed guidelines in the hospital due the lack of national guidelines and protocols for the administration of biologics for the management of IBD [31].

The Chi square and Fisher’s exact tests, and one-way ANOVA were used as appropriate to compare the utilization rates of biologics and non-biologics across CD and UC patients. All statistical analyses were conducted using SAS^®^ version 9.2 statistical software (SAS Institute Inc., Cary, NC, USA).

## Results

The present study analyzed the data of 507 IBD patients, of which 258 were affected by CD, and 249 by UC. Their baseline demographic and clinical characteristics are summarized in Table 1. The average age of the patients was 35.96 years, and 48.13% were male. Although, 80% of patients did not have any comorbidities, 63% were on polypharmacy taking four or more medications. These variables were comparable between the CD and UC groups.

**Table 1:** Baseline characteristics of IBD patients

Characteristic	CD (N = 258)	UC (N = 249)	Total (N = 507)
Age, years	33.99 ± 13.18	37.97 ± 15.25	35.96 ± 7.09
Gender			
Male, n (%)	133 (54.51)	111 (45.49)	244 (48.13)
Female, n (%)	125 (47.53)	138 (52.47)	263 (51.87)
Comorbidity, n (%)			
0	225 (87.21)	195 (78.31)	420 (82.84)
1–2	29 (11.24)	46 (18.47)	75 (14.79)
≥ 3	4 (1.55)	8 (3.21)	12 (2.37)
Medication, n (%)			
1–3	107 (41.47)	81 (32.53)	188 (37.08)
4–6	101 (39.15)	103 (41.37)	204 (40.24)
≥ 7	50 (19.38)	65 (26.10)	115 (22.68)

*CD* Crohn’s disease, *UC* ulcerative colitis

Table 2 lists all biologic and non-biologic IBD medications prescribed and dispensed for the CD and UC patients. The most commonly used biologic drugs were infliximab and adalimumab. They were prescribed more often for CD patients (infliximab: 36.82% of CD cases and 11.24% of UC cases, adalimumab: 20.54% of CD cases and 9.64% of UC cases), and this difference was statistically significant (*P *< 0.01). The other four biologics, certolizumab, golimumab, ustekinumab, and vedolizumab, were prescribed less frequently, for a total of 4.66% and 1.20% of CD and UC patients, respectively.

**Table 2:** The utilization of each biologic and non-biologic drug

Medication	CD (N = 258)	UC (N = 249)	*P*-value	Total (N = 507)
Biological agent, n (%)				
Infliximab	95 (36.82)	28 (11.24)	< 0.01*	123 (24.26)
Adalimumab	53 (20.54)	24 (9.64)	< 0.01*	77 (15.19)
Certolizumab	6 (2.33)	0	0.03	6 (1.18)
Golimumab	0	1 (0.40)	0.49	1 (0.20)
Ustekinumab	4 (1.55)	0	0.12	4 (0.79)
Vedolizumab	2 (0.78)	2 (0.80)	1	4 (0.79)
Non-biological agents, n (%)				
Azathioprine	185 (71.71)	100 (40.16)	< 0.01*	285 (56.21)
Mercaptopurine	0	3 (1.20)	0.12	3 (0.59)
Methotrexate	7 (2.71)	0	0.02	7 (1.38)
Sulfasalazine	3 (1.16)	3 (1.20)	1	6 (1.18)
Mesalazine	37 (14.34)	208 (83.53)	< 0.01*	245 (48.32)
Prednisone	0	0	–	0
Prednisolone	6 (2.33)	12 (4.82)	0.13	18 (3.55)
Hydrocortisone	3 (1.16)	3 (1.20)	1	6 (1.18)
Budesonide	9 (3.49)	0	0.01*	9 (1.78)
Corticosteroids	18 (6.98)	15 (6.02)	0.66	33 (6.51)
Metronidazole	12 (4.65)	4 (1.61)	0.03*	16 (3.16)
Ciprofloxacin	5 (1.94)	4 (1.60)	0.45	9 (1.78)

*Indicates statistically significant difference

Azathioprine was the most frequently prescribed non-biologic for CD patients (71.71% vs. 40.16% for UC, *P *< 0.01) and mesalazine was the most frequently prescribed non-biologic for UC patients (85.53% vs.14.34% for CD, *P *< 0. 01). The remaining 10 medications were prescribed less frequently, with a total of 24.92% and 17.65% of CD and UC patients, respectively, receiving these non-biologics.

**Fig. 1:**
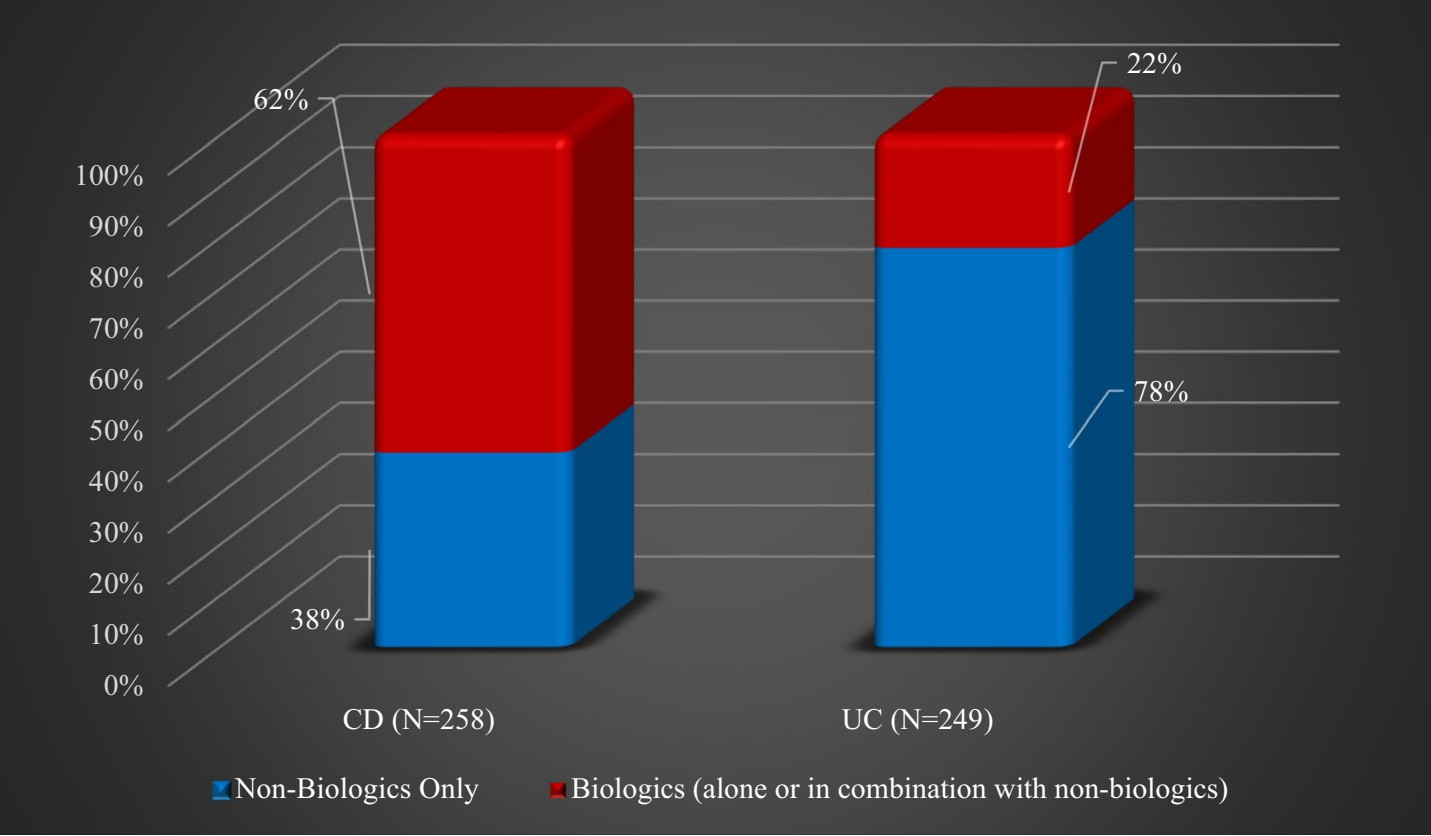
Utilization of biologic and non-biologic IBD medications

It should be noted that in most cases of IBD, a combination of drugs was employed. As indicated in Table 3, Infliximab was used in only in 6.20% of CD patients as a single medication, and in 29.84% in combination with non-biologics, most frequently with azathioprine (26.74%). Adalimumab alone was prescribed for 8.53% of CD patients and 1.20% of UC patients, but in combination with non-biologics, it was administered to 12.02% of CD patients and 8.42% of UC patients. Among the most commonly used non-biologics, azathioprine was prescribed as the only drug to 24.81% of CD patients and 2.41% of UC patients, but in combination with other non-biologics and biologics to 46.91% CD patients and 36.94% of UC patients. Mesalazine was prescribed as the only drug to 3.49% of CD patients and 45.38% of UC patients, but in combination with other non-biologics and biologics to 10.86% of CD patients and 38.14% of UC patients. The use of the azathioprine/infliximab and azathioprine/adalimumab combinations was more frequent in the CD patients (*P *< 0.01), while the use of azathioprine/mesalazine and azathioprine/mesalazine/adalimumab combinations was more frequent in the UC group (*P *< 0.01). Figure 1. illustrates the use of the two categories of drugs in both types of IBD. In comparison with biologic medications, the use of non-biologics was more prevalent in UC patients.

**Table 3:** Treatment regimens for CD and UC patients

	CD (N = 258)	UC (N = 249)	*P*-value	Antibiotics	Corticosteroids
Prescribed medications^a^, n (%)					
None	8 (3.10)	17 (6.83)	0.04*	1 (0.20)	2 (0.39)
AZA + INFX	69 (26.74)	9 (3.61)	< 0.01*	7 (1.38)	4 (0.79)
AZA	64 (24.81)	6 (2.41)	< 0.01*	2 (0.39)	4 (0.79)
AZA + ADA	23 (8.91)	2 (0.80)	< 0.01*	1 (0.20)	0
ADA	22 (8.53)	3 (1.20)	< 0.01*	1 (0.20)	1 (0.20)
INFX	18 (6.20)	0	< 0.01*	1 (0.20)	2 (0.40)
AZA + mesalazine	14 (5.43)	55 (22.09)	< 0.01*	2 (0.39)	2 (0.39)
Mesalazine	9 (3.49)	113 (45.38)	< 0.01*	0	7 (1.38)
AZA + mesalazine + INFX	4 (1.55)	11 (4.42)	0.07	0	0
AZA + mesalazine + ADA	3 (1.16)	15 (6.02)	< 0.01	0	1 (0.20)
AZA + SSZ	2 (0.78)	0	0.50	0	0
Mesalazine + INFX	3 (1.16)	7 (2.81)	0.17	1 (0.20)	2 (0.39)
Mesalazine + SSZ + UST	1 (0.39)	0	1	0	1(0.20)
Mesalazine + MTX + AZA	1 (0.39)	0	1	0	0
ADA + mesalazine	2 (0.78)	3 (1.20)	1	0	1 (0.20)
Mesalazine + AZA + SSZ	0	1 (0.40)	0.49	0	0
AZA + SSZ + INF	0	1 (0.40)	0.49	0	0
Certolizumab + MTX	1 (0.39)	0	1	0	1 (0.20)
ADA + Mesalazine + 6MP	0	1 (0.40)	0.49	0	0
AZA + certolizumab	2 (0.78)	0	0.50	0	0
ADA + MTX	2 (0.78)	0	0.50	0	0
AZA + vedolizumab	1 (0.39)	0	1	0	1 (0.20)
Certolizumab	3 (1.16)	0	0.25	0	0
Ustekinumab	2 (0.78)	0	1	0	1 (0.20)
AZA + MTX + ADA	1 (0.39)	0	1	0	0
AZA + UST	1 (0.39)	0	1	0	0
Vedolizumab + MTX	1 (0.39)	0	1	1 (0.20)	1 (0.20)
AZA + mesalazine + golimumab	0	1 (0.40)	0.49	0	0
6MP + mesalazine + vedolizumab	0	1 (0.40)	0.49	1 (0.20)	1 (0.20)
INFX + MTX	1 (0.39)	0	1	0	0
SSZ	0	1 (0.40)	0.49	0	0
Vedolizumab	0	1 (0.40)	0.49	0	0
Golimumab	0	0	–	0	0
6MP	0	1 (0.40)	0.49	0	0

*Indicates statistically significant difference

^a^*AZA* azathioprine, *INFX* infliximab, *ADA* adalimumab, *SSZ* sulfasalazine, *UST* ustekinumab, *MTX* methotrexate, *6MP* 6-mercaptopurine

Based on the DDD and prices of the medications retrieved from the SFDA database, the annual acquisition cost of biologic and non-biologic agents was calculated (Table 4). The two most commonly used biologic drugs, infliximab and adalimumab, carried the annual acquisition costs of 6023 and 16,258 USD, respectively. The least expensive biologic treatment could be provided with ustekinumab (4470 USD), while the annual cost of Vedolizumab was the highest (16,730). Azathioprine, the most commonly prescribed drug for patients with CD costs around 250 USD per year. Mesalazine, the most frequently prescribed non-biologic for UC patients, carries an annual cost of 426 USD for the oral formulation, and 977 USD for suppositories. However, IBD medications associated with much lower costs were also identified. The annual supply of the parenteral dosage form of prednisolone costs 189 USD.

**Table 4:** Estimates of annual acquisition cost of biologic and non-biologic agents

Medication	DDD^a^	Adm. R^a^	Price (US Dollars)	Annual cost^d^ (US Dollars)
Biological agents				
Infliximab	3.75 mg	Parenteral	16.50	6023
Adalimumab	2.9 mg	Parenteral	44.54	16,258
Certolizumab	14 mg	Parenteral	37.98	13,865
Golimumab	1.66 mg	Parenteral	21.99	8025
Ustekinumab	0.54 mg	Parenteral	12.25	4470
Vedolizumab	5.4 mg	Parenteral	45.84	16,730
Non-biological agents				
Azathioprine	0.15 g	Oral	0.69	254.64
Mercaptopurine	105 mg^b^	Oral	1.12	408.8
Methotrexate	2.5 mg	Oral	1.44	526.82
		Parenteral	0.24	87.41
Sulfasalazine	2 g	Oral	0.30	110.37
Mesalazine	1.5 g	Oral	1.16	425.69
		Rectal	2.67	976.98
Prednisone	10 mg	Oral	0.04	16.06
Prednisolone	10 mg	Oral	0.185	67.64
		Parenteral	0.52	189
Hydrocortisone	30 mg	Oral	0.12	45.33
		Parenteral	0.65	237
Budesonide	9 mg	Oral	^c^	^c^
Metronidazole	2 g	Oral	0.37	138.21
	1.5 g	Parenteral	6.51	2378.88
Ciprofloxacin	1 g	Oral	1.24	455.52
	0.5 g	Parenteral	4.19	1529

^*a*^*DDD* defined daily dose, *Adm. R* route of administration

^b^Calculated from usual maintenance dose 1–2.5 mg/kg

^c^The price of that specific dosage form is not available in the SFDA drug list

^d^Rounded to the nearest dollar

The administration of biologics is associated with additional costs resulting from the cost of intravenous administration (when applicable), and the cost of laboratory tests required to assure the safety of each medication. These expenses, itemized in Table 5, increase the annual expenses by a minimum of 1031 USD for adalimumab, certolizumab, and golimumab, and to a maximum of 1882 USD for Infliximab. The lowest relative increase was calculated for adalimumab (6.3%, from 16,258 to 17,289 USD), and the highest for ustekinumab (24.6%, from 4471 to 5572 USD).

**Table 5:** Breakdown of annual administration cost of biologics

	Cost categories	Biological agents					
		Infliximab	Adalimumab	Certolizumab	Golimumab	Ustekinumab	Vedolizumab
Treatment course	Defined daily dose (DDD)	3.75 mg	2.9 mg	14 mg	1.66 mg	0.54 mg	5.4 mg
	Acquisition price per day	16.5 USD	44.54 USD	37.98 USD	21.98 USD	12.25 USD	45.83 USD
	Route of administration	IV	SubQ	SubQ	SubQ	1st dose: IV Other doses: SubQ	IV
	Infusion time	1st dose = 3 h other doses = 2 h	N/A	N/A	N/A	1 h	30 min
	The doses per year	1st year= 9 doses Next year = 7 doses	1st year= 27 doses Next year = 26 doses	1st year=15 doses Next year = 13 doses	1st year= 14 doses Next year = 13 doses	1st year= 6 doses Next year = 6 doses.	1st year= 9 doses Next year = 6 doses
Lab tests (all at baseline, and CBC as indicated for each med.)	Tuberculin Test	46.13 USD	46.13 USD	46.13 USD	46.13 USD	46.13 USD	46.13 USD
	Chest X-ray	53.33 USD	53.33 USD	53.33 USD	53.33 USD	53.33 USD	53.33 USD
	HIV serology	170.67 USD	170.67 USD	170.67 USD	170.67 USD	170.67 USD	170.67 USD
	Hepatitis B and C serology	72.80 USD	72.80 USD	72.80 USD	72.80 USD	72.80 USD	72.80 USD
	VZV serology	114.93 USD	114.93 USD	114.93 USD	114.93 USD	114.93 USD	114.93 USD
	HSV serology	120 USD	120 USD	120 USD	120 USD	120 USD	120 USD
	CBC	26.67 USD	26.67 USD	26.67 USD	26.67 USD	26.67 USD	26.67 USD
	U&E, LFT, ESR, serum albumin and CRP	320 USD	320 USD	320 USD	320 USD	320 USD	320 USD

		CBC at every administration	CBC every 3 months	CBC every 3 months	CBC every 3 months	CBC every 3 months	CBC at every administration
Pre-medication (at baseline and at every administration)	Intravenous Infusion procedure	53.33 USD	0 USD	0 USD	0 USD	53.33 USD	53.33 USD
	Normal saline	0.93 USD	0 USD	0 USD	0 USD	0.93 USD	0.93 USD
	Hydrocortisone IV injection of 100 mg	2.17 USD	0 USD	0 USD	0 USD	2.17 USD	2.17 USD
	Chlorpheniramine IV injection of 10 mg	0.32 USD	0 USD	0 USD	0 USD	0.32 USD	0.32 USD
	Nursing fee per hour^a^	1st dose = 40 USD Other doses = 26.67 USD	0 USD	0 USD	0 USD	13.33 USD	6.66 USD
Annual acquisition cost^b^	Annual acquisition cost based on DDD	6023 USD	16,258 USD	13,865 USD	8025 USD	4471 USD	16,730 USD
	Lab tests’ costs	1138 USD	1031 USD	1031 USD	1031 USD	1031 USD	1138 USD
	IV Pre-medication costs and nursing fee	744 USD	0 USD	0 USD	0 USD	70 USD	556 USD
	Total cost for the first year of treatment	7905 USD	17,289 USD	14,896 USD	9056 USD	5572 USD	18,424 USD

*IV* intravenous route, *SubQ* subcutaneous route, *DDD* defined daily dose, *N/A* not applicable, *CBC* complete blood count, *HIV* human immunodeficiency virus, *VZV* Varicella zoster virus, *HSV* Herpes simplex virus, *U&E* Urea and electrolytes, *LFT* liver function test, *ESR* erythrocyte sedimentation rate, *CRP* C-reactive protein

^a^Nursing fee per hour is 13.33 USD

^b^Rounded to the nearest dollar

## Discussion

IBD is one of the most expensive to treat gastrointestinal disorder, even if the cost of medications is only considered [32, 33], and its increasing incidence and prevalence outside of the Western world constitute a significant challenge to healthcare systems [34]. The data collected in the present study document the high cost of IBD treatment in Saudi Arabia, which is comparable to that seen in Western countries [25, 26, 28]. Among this study sample, which included Saudi patients with IBD, the high financial burden was seen both in the patients diagnosed with CD, of which 68% were treated with biologics alone or in combination with non-biologics, as well as in UC patients, of which 78% were treated with non-biologics exclusively. However, patients with CD had generally higher utilization rate of medications particularly biologic drugs which is consistent with previously published research [13, 23, 24, 35].

The least expensive medications used in the treatment of IBD have been identified in the group of non-biologic drugs designed for parenteral administration, such as methotrexate, prednisolone, hydrocortisone, and ciprofloxacin, with the annual cost below 2000 USD. In contrast, the annual expenditures associated with the administration of biologics, comprising the acquisition costs of medications as well as the costs of laboratory tests, intravenous pre-medication administration, and nursing, were 5572 USD for ustekinumab which was the least expensive biologic, and exceeded $17,000 for adalimumab and vedolizumab. However, the expected increase in the financial burden of IBD might be mitigated to some extent by the predicted lower price of biosimilars when this new group of medications is introduced [36].

The necessity to decrease the dependence on oil revenue inspired the development of the transformative economic program for the country, Saudi Vision 2030 [37], which drives sweeping changes in the economic and cultural landscape of Saudi Arabia. Healthcare reform is an integral part of this program. The move from the economy led by the government to free market-based economy will affect the way medical services are delivered to the residents in the Kingdom. This transformation aims to improve the quality of health care and to expand the privatization of governmental services. The operational plan includes increasing the participation of private insurance companies in financing health care services [38]. Since the limited access to medications has been indicated as one of the causes of dissatisfaction with the current public sector healthcare services, and the support for privatization is conditional on reversing this situation [39]. It is possible, however, that the planned reform will facilitate patient access to more effective and expensive IBD medications, including both non-biologics and biologics.

The high cost of biologics noted in the present study may be reduced by the introduction of biosimilars, i.e., biological molecules that are highly similar to the originally patented reference biologics. Biosimilars are already available for infliximab, and more biosimilars are expected to enter the market as the original biologics lose the protection of their market exclusivity. Moreover, it is estimated that the use of biosimilars could result in savings of up to $22 billion annually in the European Union and the United States alone [40]. It can be expected that similar savings could be achieved in Saudi Arabia, reducing the strain on health care budgets, whether governmental or private. Lowering IBD treatment costs by substituting biologics with biosimilars may have an additional benefit for the Saudi population since the proportion of people over 65 years of age is expected to double in the next decade putting them at higher risk of having different autoimmune diseases such as rheumatoid arthritis in which the use of biologics to manage such diseases is common and well-established [41, 42]. However, the listed public prices of some registered biosimilars in the SFDA drug prices database are largely similar to the originators such as the case with infliximab. Moreover, the listed drug prices in the SFDA drug prices database do not reflect the real purchasing prices that different health care institutions, either governmental or private, buy their medications in. In many instances, the offered prices of biologics (originators) in different pharmaceutical tenders are lower than their biosimilar counterparts. Therefore, for biosimilars to be cost effective in Saudi Arabia their prices need to be reduced significantly compared to their originators, otherwise, the cost of biologics will remain a significant challenge for the healthcare system.

It should be noted that while biologics tend to yield better outcomes than non-biologics, they overall carry a higher utilization cost and may not be considered cost-effective, particularly when used as a maintenance therapy [43]. In addition, the use of biologics instead of non-biologics may delay the need to perform surgical interventions, particularly in patients suffering from UC [44]. Furthermore, combination therapies using biologics antagonizing TNF-α and corticosteroids are associated with higher risk of serious infections [45]. All these factors will undoubtedly be scrutinized by private insurance companies, and may impact their decision on which IBD medications are covered by their different insurance plans. Therefore, it is imperative that cost effective pharmacologic strategies are identified which consider clinical benefits and lifetime costs. For these approaches to be developed, they must also account for the long-term sequelae of inhibition of TNF-α signaling, a major mechanism of action of biologics used in IBD, which remain to be seen [27]. Thus, a significant research effort is necessary before conclusive evidence-based recommendations for the use of biologics in the treatment of IBD among Saudi patients are reached.

Although this study is the first to the best of our knowledge to report the utilization patterns and annual acquisition costs of different biologic and non-biologic drugs used in the management of IBD in Saudi Arabia, several limitations of the findings must be acknowledged. First, the study was a single center cross-sectional study, which limits the generalizability of its findings despite the fact that the hospital in which the patients were recruited from was a tertiary referral hospital where many patients with IBD in Riyadh and elsewhere in the Kingdom are referred to. Moreover, multiple challenges and obstacles in locating the needed data were faced during the data collection process due to the poor documentation of data particularly medications. Many important variables such as the severity and duration of illness were not found in either the paper or electronic based medical records. The availability of such data may have explain the differences in the treatment regimens among the studied cohort of IBD patients. Additionally, the use of DDD to calculate the annual utilization cost of different biologic and non-biologic drugs may not provide an accurate estimate of the actual utilization cost since the DDD provides a rough estimate for the consumption of medications for adults [30]. However, the variable dosage of biologic and non-biologic drugs based on patient weight as well as other variables such as the renal and hepatic functions makes the use of DDD to estimate the daily or annual consumption of different medications and eventually their annual utilization costs more practical to provide a rough estimate about the cost of therapy for other patients in Saudi Arabia who were not part of this study. Furthermore, most of the patients were on combination therapy and the costs presented were for each IBD medication individually. Therefore, it is expected that the cost of IBD medications for each patient is significantly higher. Finally, establishing a national IBD patient registry to evaluate the cost and efficacy of different therapeutic interventions in the management of IBD is important.

## Conclusions

The overall utilization rate of biologics in the management of IBD has significantly increased over the last decade putting increasing pressure on the national health care system in Saudi Arabia. Therefore, exploring the cost effectiveness of different options such as switching to biosimilars is warranted.

## Data Availability

The datasets during and/or analyzed during the current study available from the corresponding author on reasonable request.
